# Neurofilament Light Chain from Neuronally Derived Extracellular Vesicles in Differentiating Parkinson’s Disease from Essential Tremor with Resting Tremor

**DOI:** 10.1007/s12035-025-05285-7

**Published:** 2025-11-11

**Authors:** Selena Mimmi, Costanza Maria Cristiani, Mariagrazia Talarico, Anna Maria Tolomeo, Elvira Immacolata Parrotta, Luana Scaramuzzino, Valentina Crapella, Elisabetta Pingitore, Enrico Iaccino, Giovanni Cuda, Aldo Quattrone, Andrea Quattrone

**Affiliations:** 1 Neuroscience Research Center, University “Magna Graecia”, Catanzaro, Italy; 2 Department of Experimental and Clinical Medicine, University “Magna Graecia”, Catanzaro, Italy; 3 Institute of Pediatric Research Città Della Speranza, Padua, Italy; 4 Department of Cardiac, Thoracic and Vascular Science and Public Health, University of Padova, Padua, Italy; 5 Laboratory of Stem Cells, Department of Medical and Surgical Sciences, University “Magna Graecia”, Catanzaro, Italy; 6 Institute of Neurology, Department of Medical and Surgical Sciences, University “Magna Graecia”, Catanzaro, Italy

**Keywords:** Parkinson’s disease, Essential tremor with resting tremor, Neuronally derived extracellular vesicles, Neurofilament light chain

## Abstract

Parkinson’s disease (PD) and essential tremor with resting tremor (rET) show a remarkable symptom overlap that complicates the clinical differentiation and highlights the need for easily assessable diagnostic biomarkers. Neuronally derived extracellular vesicles (NDEVs) have been suggested as a peripheral substrate providing insights on the central nervous system pathology. Here, we assessed the accuracy of NDEV neurofilament light chain (Nf-L) levels in discriminating between PD and rET patients. Thirty-three PD and 13 rET patients and 30 healthy controls (HC) were consecutively enrolled in the study. NDEVs were obtained from serum through size exclusion chromatography followed by immunocapture based on L1CAM expression. NDEV Nf-L levels were quantified by ELISA on Ella Platform. Receiver operator characteristic (ROC) analysis was performed to investigate the NDEV Nf-L classification performance. Associations between NDEV Nf-L and demographical or clinical variables were investigated with Spearman’s method. NDEV Nf-L levels were significantly higher in PD compared to rET patients and HC, and achieved an excellent classification performance in distinguishing PD from both rET and HC (AUC: 0.902 and 0.987, respectively). No differences were observed between rET and HC. A significant negative association between NDEV Nf-L and sex was detected in rET patients. Our pilot study suggests that NDEV Nf-L might be a useful blood-derived biomarker helping the differential diagnosis between PD and rET patients. Future studies are needed to validate these findings in larger independent cohorts.

**Supplementary Information:**

The online version contains supplementary material available at 10.1007/s12035-025-05285-7.

## Introduction

Among tremor disorders, essential tremor (ET) and Parkinson’s disease (PD) are the most common forms observed in the elderly [1]. PD is characterized by the loss of dopaminergic neurons within the substantia nigra compacta, particularly at ventrolateral level, together with widespread presence of intracytoplasmic Lewy bodies (LBs) and dystrophic Lewy neurites, composed by α-synuclein aggregates [2]. Conversely, ET may be characterized by loss and morphological alterations of Purkinje cells within the cerebellum [3]. However, despite the differences in neurons and brain areas involved, ET and PD show remarkable symptom overlap. Indeed, although ET is mainly characterized by bilateral action tremor while unilateral resting tremor is predominant in PD [4, 5], the co-occurrence of the two types of tremor is frequent in both PD and ET patients [1, 3]. Particularly, resting tremor can occur in up to 18% of ET (rET) patients [6], with a clinical picture resembling PD, which complicates the clinical diagnosis. Currently, the gold standard for differentiating PD from ET is the single photon emission computed tomography with 123I-ioflupane (DaTscan) [7], but it is an invasive, expensive and time-consuming procedure. This highlights the need for easily accessible biomarkers to be applied in clinical practice. Among them, molecular blood-based biomarkers would fulfil the requirement of accessibility, non-invasiveness and standardization.

The light chain of neurofilaments (Nf-L) is one of the most widely investigated fluid biomarkers in the context of parkinsonism, showing modestly increased peripheral levels in PD patients compared to healthy controls (HC) and a markedly pronounced elevation in atypical parkinsonian syndromes compared to PD [8–10]. On the other hand, Nf-L has been poorly investigated in ET, providing conflicting results [10–13]. Recent studies suggested that neuronally derived extracellular vesicles (NDEVs), which are extracellular vesicles (EVs) released by neurons and containing proteins and other biomolecules reflecting the state and composition of the parent cell, would provide a more detailed picture of the pathological processes occurring within CNS compared to molecules directly found in serum or plasma [14]. As an example, α-synuclein measured in serum or plasma showed inconsistency as PD biomarker [15–17], while its quantification within neuronally derived extracellular vesicles (NDEVs) provided more consistent results [18–21]. Since NDEVs can cross the blood–brain barrier, immunocapturing using an antibody against the neuronal protein L1CAM can be employed to easily isolate them from blood [22].

Notably, NDEV protein content has been poorly investigated in the context of ET [23, 24], and no data exist on Nf-L in ET with resting tremor (rET). In this pilot study, we specifically focused on PD and rET, investigating Nf-L in NDEVs for distinguishing between these two tremulous disorders.

## Methods

### Participants

Thirty-three PD and 13 rET patients were consecutively enrolled in the study. All patients were examined by movement disorder specialists, and the diagnoses were performed in accordance with international diagnostic criteria [4, 5]. Disease severity in PD patients was scored by the MDS—Unified Parkinson’s disease rating scale and Hoehn and Yahr (HY) rating scale in practical off-state, while Fahn-Tolosa-Marín tremor rating scale was used for rET patients. A 3 T brain MRI and DaTscan were performed in all patients, and surface electromyographic assessment was employed to confirm resting tremor in rET [25, 26]. Clinical and MRI abnormalities suggestive of other diseases, lacunar infarctions in the basal ganglia, diffuse brain vascular lesions, current or past use of medications known to cause or exacerbate tremor were considered exclusion criteria. Thirty subjects unaffected by any neurological disorder and without affected close relatives were enrolled as healthy controls (HC).

### EVs Characterization, NDEVs Isolation and Nf-L Quantification

From each enrolled subject, serum sample was collected in BD Vacutainer™SSTTM Serum Separation Tubes (BD, Franklin Lakes, NJ, USA) between 9 a.m. and 12 p.m. Within 30 min, samples were centrifuged at 3000 rpm at 20 °C for 10 min, aliquoted and stored at − 80 °C until use. Two-step size exclusion chromatography was employed to isolate EVs [27]. In brief, 500 μL of serum was loaded into qEVoriginal/35 nm columns (Izon Science Ltd., Christchurch, New Zealand). Following the manufacturer’s protocol, a homogeneous population of particles primarily sized below 150 nm was obtained, as confirmed by tunable resistive pulse sensing (tRPS) technology (EXOID, Izon Science Ltd.) on NP100 membrane. CPC100 calibration solution (110 nm mean carboxylate polystyrene beads) was used to standardize particle concentration A Tecnai G2 (FEI) transmission electron microscope (TEM) operating at 100 kV was used for morphological analysis [28], confirming that the obtained particles were “small EVs”, with a size range of 30–150 nm [29].

Immunoaffinity capture employing L1CAM-decorated streptavidin magnetic beads was used to isolate NDEVs from the whole EV population. A normal IgG was used as negative control. EVs were resuspended in 500 μL of PBS 1X and incubated overnight at 4 °C with the capture system, composed of 100 μL of streptavidin magnetic beads (Promega Italia Srl, Milan, Italy) conjugated with 7 μg of biotinylated-anti L1CAM antibody, clone n.3H7B9 (Proteintech, Manchester, UK), according to the manufacturer’s instructions. NDEVs bound by beads were washed 3 times with PBS 1X plus with 0.05% Tween_20_ and resuspended in 500 μL of RIPA buffer (Millipore, Burlington, MA, USA) supplemented with protease and phosphatase inhibitors to extract proteins. After 1 h of incubation in ice followed by three freeze–thaw cycles (30 s at − 80 °C and then vortex), NDEV lysates were centrifuged at 12,000 g at 4 °C. Protein containing supernatants were then aliquoted and stored at − 80 °C. Overall, the same volume of serum and lysing buffer (500 μL) was used in all the samples to standardize final protein content.

A rapid BCA Protein Assay Kit (Thermo Scientific, Rockford, USA) was used to determine protein concentrations. The same amount of protein was isolated using 10% SDS-PAGE and then transferred to a nitrocellulose membrane (Thermo Scientific, Rockford, USA). Anti CD63 (EXOAB-CD63A-1—System Biosciences, LLC, Palo Alto, CA), anti CD9 (EXOAB-CD9A-1—System Biosciences, LLC, Palo Alto, CA), anti CD81 (EXOAB-CD81A-1—System Biosciences, LLC, Palo Alto, CA) and anti-L1CAM (Antibodies.com, Cambridge, UK) were incubated overnight at 4 °C. Protein bands were detected and analysed using the chemiluminescence detection system UVITEC Cambridge.

For flow cytometry assay, NDEVs bound by beads and normal IgG decorated beads were stained with anti CD63, anti CD81 and anti-L1CAM (Miltenyi Biotec, Bergisch Gladbach, Germany) overnight at 4 °C and resuspended in facs flow buffer, Samples were first analysed for physical parameters (FSC-A and SSC-A plot) and then for fluorescence intensity.

Nf-L quantification was performed by using human Nf-L Simple Plex™ assay (Bio-Techne, Minneapolis, MN, USA) on Ella™ Automated ELISA platform (Bio-Techne).

### Statistical Analysis

All the statistics was performed on IBM SPSS v29.0.1.0 software (Armonk, NY, USA). *Χ*^2^ test was applied to assess differences in sex distribution. Normal distribution of continuous variables was assessed by Shapiro–Wilk test. Based on the distribution, analysis of variance (ANOVA) followed by Bonferroni’s correction was used to assess differences in age at examination while Mann–Whitney test was used to assess differences in disease duration. To compare NDEV Nf-L between HC and patient groups, ANCOVA with sex and age as covariates followed by Tukey’s Honestly Significant Difference (HSD) test was used, while disease duration was also considered a covariate in comparing rET and PD. Receiving operating characteristics (ROC) curves were applied to assess NDEV Nf-L diagnostic value and Youden Index used to select the cutoff providing the maximum sum of sensitivity and specificity. Spearman’s test was employed to investigate correlations between NDEV Nf-L and demographical or clinical variables. All tests were two tailed, and a *p* value < 0.05 was considered significant for all the analyses.

### Protocol Approvals

The study was performed in accordance with The Declaration of Helsinki and approved by the Calabria Region Ethics Committee (under protocol code 143 on 13 May 2024). A written informed consent to study participation was obtained from all the recruited subjects, both for study participation and data publication.

## Results

### Demographic and Clinical Characteristics

The cohort included 76 participants (13 rET, 33 PD patients and 30 HC) whose demographic and clinical features are summarized in Table 1. HC showed a higher female prevalence compared to the other two groups and were younger compared to PD patients. Moreover, rET patients showed a longer disease duration compared to PD, in accordance with the earlier onset of the disorder [7].

**Table 1:** Demographic and clinical features of rET, PD patients and HC. Data are shown as mean ± SD

	rET (*n* = 13)	PD (*n* = 33)	HC (*n* = 30)	*p* value
Sex (F/M)	6/7	13/20	22/8	0.021^a^
Age (years)	64.5 ± 12.27	69.4 ± 7.29	60.2 ± 10.52	< 0.001^b,*^
Disease duration (years)	10.3 ± 9.20	4.8 ± 3.45	-	0.023^c^
MDS-UPDRS-III		20.9 ± 10.56		-
HY rating scale		1.8 ± 0.80		-
Fahn-Tolosa-Marín tremor rating scale	12.8 ± 7.38	-	-	-

*rET* essential tremor with resting, *PD* Parkinson’s disease, *HC* healthy control, *MDS-UPDRS* MDS—Unified Parkinson’s disease rating scale—pars III, *HY* Hoehn and Yahr

^a^*Χ*^2^ test

^b^ANOVA followed by Bonferroni’s correction

^c^Mann-Whitney *U* test

^*^p values after Bonferroni’s correction: PD vs. HC: < 0.001; PD vs. rET: 0.36; rET vs. HC: 0.55

### Neurofilament Light Chain in Neuronally Derived Extracellular Vesicles

**Fig. 1:**
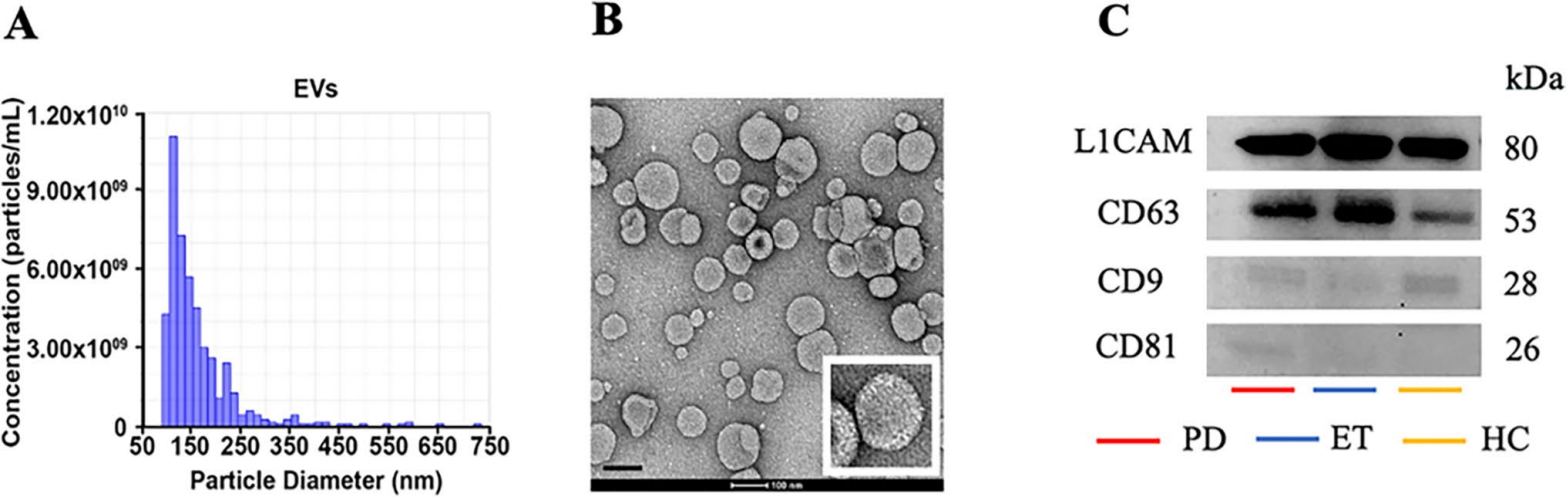
Total EVs and NDEVs characterization. Total EVs size distribution was assessed by tunable resistive pulse sensing (tRPS) technology (**A**) and transmission electron microscopy (**B**) (scale bar = 100 nm; insert shows higher-magnification image). Prior to NEDVs isolation, both approaches confirmed that the obtained vesicles fulfil the “small EVs” criteria. Western blotting analysis confirmed the NDEVs enrichment, showing the tetraspanines (CD63, CD9, CD81) as well as L1CAM in all groups in analysis (**C**)

Morphological assessment of serum nanoparticles showed that they possessed a diameter of 30–150 nm, a spherical shape and a lipid bilayer, in line with the MISEV2018 criteria for “small EVs” (Fig. 1A,B) [29]. Western blotting analysis (Fig. 1C) confirmed the NDEVs enrichment, by the evaluation of tetraspanines expression (CD63, CD9 and CD81) as well as the neuronal marker L1CAM. No EVs were detected in flow cytometry using as capture antibody a normal IgG (Supplementary Figure S1).

**Fig. 2:**
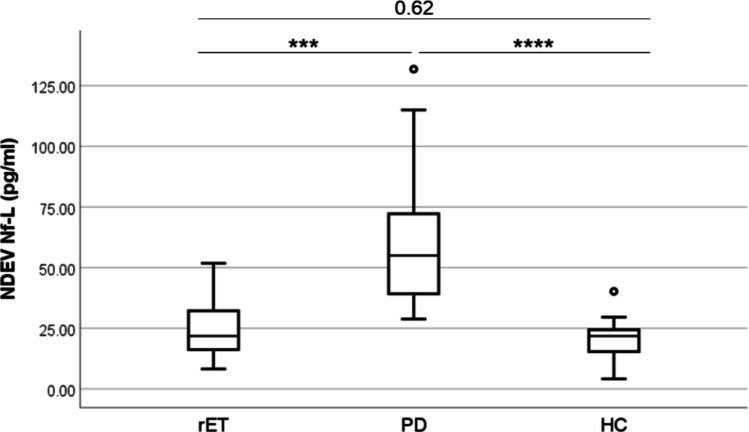
NDEV concentration of Nf-L in rET (*n* = 13), PD (*n* = 33) and HC (*n* = 30). Data are summarized as box plots, where the lower, middle and upper lines represent the 25th percentile, median and 75th percentile, respectively. Ranges are depicted by vertical lines, while moderate outliers are depicted by dots. Statistical analysis was performed by ANCOVA with sex, age (HC vs. rET vs. PD), and disease duration (rET vs. PD) as covariates, followed by Tukey’s HSD test; ****: *p* value < 0.0001, ***: *p* value = 0.001; NDEV neuronal-derived extracellular vesicle, Nf-L neurofilament light chain, rET essential tremor with resting, PD Parkinson’s disease, HC healthy control

In all the subjects, protein lysate derived by peripheral NDEVs was positive for Nf-L. In detail, NDEV Nf-L showed a significantly higher concentration in PD patients compared to both the other groups (PD vs. rET: *p* value = 0.001; PD vs. HC: *p* value < 0.0001), while NDEV Nf-L levels between ET and HC were largely overlapping (*p* value = 0.62) (Fig. 2).

Higher levels of NDEV Nf-L were associated with the female sex in rET (*p* value = 0.01), while no associations were observed with demographical or clinical features in the other groups (Table 2).

**Table 2:** Correlation analysis of NDEV Nf-L levels with demographic and clinical variables in PD and ET and with sex and age in HC

Group		Sex	Age	Disease duration	MDS-UPDRS-III	HY rating scale	FTM
rET (*n* = 13)	rho	0.66	0.04	0.41	-	-	0.01
	*p* value	*0.01*	0.91	0.16	-	-	0.98
PD (*n* = 33)	rho	0.21	0.19	− 0.32	0.17	0.11	-
	*p* value	0.26	0.29	0.07	0.35	0.55	-
HC (*n* = 30)	rho	0.13	0.16	-	-	-	-
	*p* value	0.51	0.41	-	-	-	-

*NDEV* neuronal-derived extracellular vesicle, *Nf-L* neurofilament light chain, *rET* essential tremor with resting, *PD* Parkinson’s disease, *HC* healthy control, *MDS-UPDRS* MDS—Unified Parkinson’s disease rating scale—pars III, *HY* Hoehn and Yahr, *FTM* Fahn-Tolosa-Marín tremor rating scale

## Classification Performances Between Groups

**Fig. 3:**
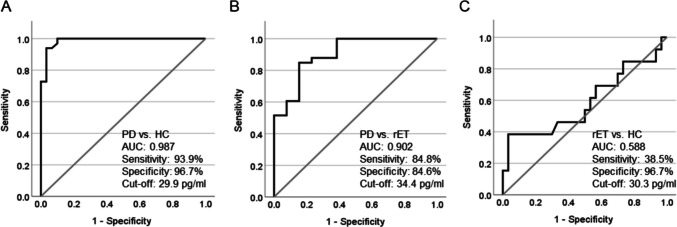
Receiver operating characteristic curves of NDEV nf-L in differentiating PD from HC (**A**), PD from rET (**B**) and rET from HC (**C**). PD Parkinson’s disease, rET essential tremor with resting, HC healthy control, AUC area under the curve

We employed receiver operating characteristic (ROC) curves to evaluate the accuracy of NDEV Nf-L in discriminating between groups. In detail, NDEV Nf-L concentration demonstrated an excellent accuracy in distinguishing PD from both HC and rET. Particularly, NDEV Nf-L levels distinguished PD from HC with an AUC of 0.987 (95% CI: 0.967–1.008), a sensitivity of 93.9% (95% CI: 80.39–98.92%) and a specificity of 96.7% (95% CI: 83.33–98.93%) (Fig. 3A), and PD from rET with an AUC of 0.902 (95% CI: 0.804–1.000), a sensitivity of 84.8% (95% CI: 69.08–93.35%) and a specificity of 84.6% (95% CI: 57.77–97.27%) (Fig. 3B).

## Discussion

In this preliminary study, we showed that Nf-L encapsulated within NDEVs were increased in PD compared to rET patients and healthy subjects and accurately distinguished PD patients from both rET and HC participants. Overall, our data indicate that NDEV Nf-L might represent a promising accessible and accurate blood-based biomarker to accurately discriminate PD from rET, holding promise for its application in clinical practice.

Since PD and ET exhibit different types of tremors [4, 5], the differential diagnosis mainly relies on clinical symptoms. However, since typical signs of PD can occur in ET and vice versa, up to one third of ET patients are misdiagnosed as PD. This is particularly relevant in the case of rET, in which patients show resting tremor, which is a core sign in PD [1]. Therefore, more specific tools to be used in clinical practice are highly needed to identify resting tremor of parkinsonian origin. Molecular biomarkers would be useful not only for differential diagnosis but also to shed light on the pathological mechanisms underlying the diseases.

Nf-L is a neuronal cytoplasmatic protein mainly expressed by myelinated axons and involved in several neuronal processes such as cytoskeleton stabilization, axonal transport, interaction with myelin proteins and mitochondria distribution. In the presence of neuro-axonal damage, Nf-L is released into the cerebrospinal fluid and then into the blood through the blood–brain barrier [30]. As shared marker of neurodegeneration, increased peripheral concentration of Nf-L has been detected in blood or cerebrospinal fluid of several neurological disorders, such as multiple sclerosis, amyotrophic lateral sclerosis, PD and atypical parkinsonisms, neurodegenerative dementias and traumatic brain injury (TBI) [9]. Conversely, blood levels of Nf-L have been found to be unchanged [10, 11] or only modestly increased [12, 13] in ET compared to control subjects. No study however focused on rET patients with resting tremor. In the current study, we explored for the first time Nf-L in neuronally derived EVs of ET patients with resting tremor, and we did not detect significant differences in NDEV Nf-L levels between rET and HC, suggesting either that neuronal damage in rET is limited compared to other disorders or that cerebellar degeneration is not associated with Nf-L release. However, the evidence that peripheral Nf-L is increased in other forms of cerebellar degeneration such as spinocerebellar ataxia [31–33] supports the first hypothesis.

While there is a wide literature regarding Nf-L directly detected in blood [9, 10], its assessment within EVs has been very limited and performed mostly in the context of traumatic brain injury, where it has been shown to be elevated and correlate with clinical symptoms and injury severity [34–36]. Only two studies investigated EV Nf-L in PD, reporting no differences between patients and controls [37, 38]. However, it should be noted that in these works Nf-L was evaluated in the whole EV population, regardless their cellular origin. In contrast, we detected a marked difference in Nf-L concentration between PD and HC by assessing the marker specifically within the neuronally derived population, suggesting that NDEVs are a more representative matrix of CNS physiopathology compared to the general EV population.

In addition, we observed a significant correlation between sex and NDEV Nf-L in rET patients, indicating that the biomarker tended to be more elevated in females compared to males in these subjects. However, we did not detect such an association in the other groups, and there is no convincing evidence on differences in blood Nf-L levels between sexes [9]. Therefore, it is possible that the correlation we detected was due to the small number of rET patients.

Our study has several strengths. First, it is the first work investigating Nf-L levels within NDEVs instead of using the whole peripheral EV population. Second, we specifically focused on Nf-L levels to differentiate PD from ET with resting tremor, while previous studies assessed this biomarker in the classical ET, without differentiating for the presence of resting tremor [10–13]. Third, DaTscan, the gold standard procedure for differential diagnosis between PD and rET, was performed in all patients, noticeably minimizing the risk of misdiagnoses.

Some limitations must be considered in this study. First, we enrolled a limited number of rET patients due to the modest frequency of this syndrome [6], which did not allow for further stratification of patients based on other clinical parameters such as disease duration. Consequently, we do not know whether the difference in NDEV Nf-L levels between PD and rET occurs early in the course of the disease or is a later phenomenon. Therefore, larger studies are needed either to confirm our results and to assess the possibility of using NDEV Nf-L as early prognostic discriminative biomarker. Second, some misdiagnoses of PD patients with other parkinsonisms showing nigrostriatal degeneration might have occurred. Indeed, although PD diagnosis was based on international criteria [4, 5], made by movement disorder specialists and supported by DaTscan, post-mortem neuropathological confirmation is missing. Third, due to the cross-sectional design of our study, we have no cues regarding the capability of NDEV Nf-L levels in predicting the course of the diseases. This would be of particular importance for rET patients, since ET is a well-known risk factor for PD, with ET patients showing a 4–5 times higher risk of developing PD compared to the general population [39]. Therefore, longitudinal studies will be warranted to assess the utility of NDEV Nf-L in predicting PD development in rET. Lastly, NDEVs were isolated based on L1CAM, whose specificity raised some concerns since it can be detected also in many types of neuronal and non-neuronal cells [40]. Additionally, L1CAM exists in multiple forms, possibly negatively affecting the specificity [41]. As a consequence, L1CAM-positive EVs should be regarded as a CNS-enriched than a pure population.

## Conclusions

We analysed for the first time NDEV Nf-L in PD and rET patients, showing that it accurately distinguished these two neurodegenerative diseases. While this study provides promising initial evidence, we acknowledge that technical validation and linearity data for the Nf-L assay were not included in this exploratory analysis. Future studies will include full analytical validation following FDA/EMA guidelines. Furthermore, we are fully aware that the cohort size and imbalance limit the statistical power, especially in subgroup analyses. Larger, multicenter studies including clinically relevant disease controls (e.g., MSA, PSP) are warranted to validate these findings. If validated in larger cohort, this biomarker may represent a useful tool supporting differential diagnosis in clinical practice.

## Supplementary Information

Below is the link to the electronic supplementary material.

ESM 1 Supplementary Material 1 (DOCX 131 KB)

ESM 2 Supplementary 2 (PNG 196 KB)

High Resolution Image (TIF 10.4 MB)

## Data Availability

Due to privacy restingrictions, the data supporting the results of this study are not publicly available and can be reasonably requested from the corresponding author.
