# Supplementary material for: Neurofilament Light Chain from Neuronally Derived Extracellular Vesicles in Differentiating Parkinson’s Disease from Essential Tremor with Resting Tremor
Source: Mol Neurobiol. 2025 Nov 11;63(1):24. doi: 10.1007/s12035-025-05285-7 (PMC12605399; doi:10.1007/s12035-025-05285-7)
Supplement: Supplementary file 1 — Supplementary Material 1 (DOCX 131 KB) [file 12035_2025_5285_MOESM1_ESM.docx]

**Neurofilament Light Chain from Neuronally-Derived Extracellular Vesicles in differentiating Parkinson’s Disease from Essential Tremor with Resting Tremor**

Selena Mimmi^1,°^ (<https://orcid.org/0000-0003-2187-9176>) , Costanza Maria Cristiani^1,°^ (<https://orcid.org/0000-0002-7811-2083>), Mariagrazia Talarico^2,°^ (<https://orcid.org/0000-0002-7439-3973>), Anna Maria Tolomeo^3,4^ (<https://orcid.org/0000-0001-5077-0845>), Elvira Immacolata Parrotta^5^ (<https://orcid.org/0000-0001-8145-3623>), Luana Scaramuzzino^1^ (<https://orcid.org/0000-0003-3239-3745>), Valentina Crapella^2^, (<https://orcid.org/0000-0002-8334-0005>), Elisabetta Pingitore^2^ (<https://orcid.org/0009-0002-9023-710X>), Enrico Iaccino^2,*,^^ (<https://orcid.org/0000-0002-3565-7817>), Giovanni Cuda^2,^^ (<https://orcid.org/0000-0001-6313-1866>), Aldo Quattrone^1,^^,(<https://orcid.org/0000-0003-2001-957X>), Andrea Quattrone^1,6,*,^^ (<https://orcid.org/0000-0003-2071-2083>)

^1^ Neuroscience Research Center, University “Magna Graecia”, Catanzaro, Italy

^2^ Department of Experimental and Clinical Medicine, University “Magna Graecia”, Catanzaro, Italy

^3^ Institute of Pediatric Research Città della Speranza, Padua, Italy

^4^ Department of Cardiac, Thoracic and Vascular Science and Public Health, University of Padova,

Padua, Italy

^5^ Laboratory of Stem Cells, Department of Medical and Surgical Sciences, University "Magna Graecia", Catanzaro, Italy.

^6^ Institute of Neurology, Department of Medical and Surgical Sciences, University “Magna Graecia”, Catanzaro, Italy

^*^ Correspondence: iaccino@unicz.it (E.I.); [an.quattrone@unicz.it](mailto:an.quattrone@unicz.it) (A.Q.)

^°^ These authors shared the first authorship

^ These senior authors equally contributed to this work

**Supplementary Figures**

**
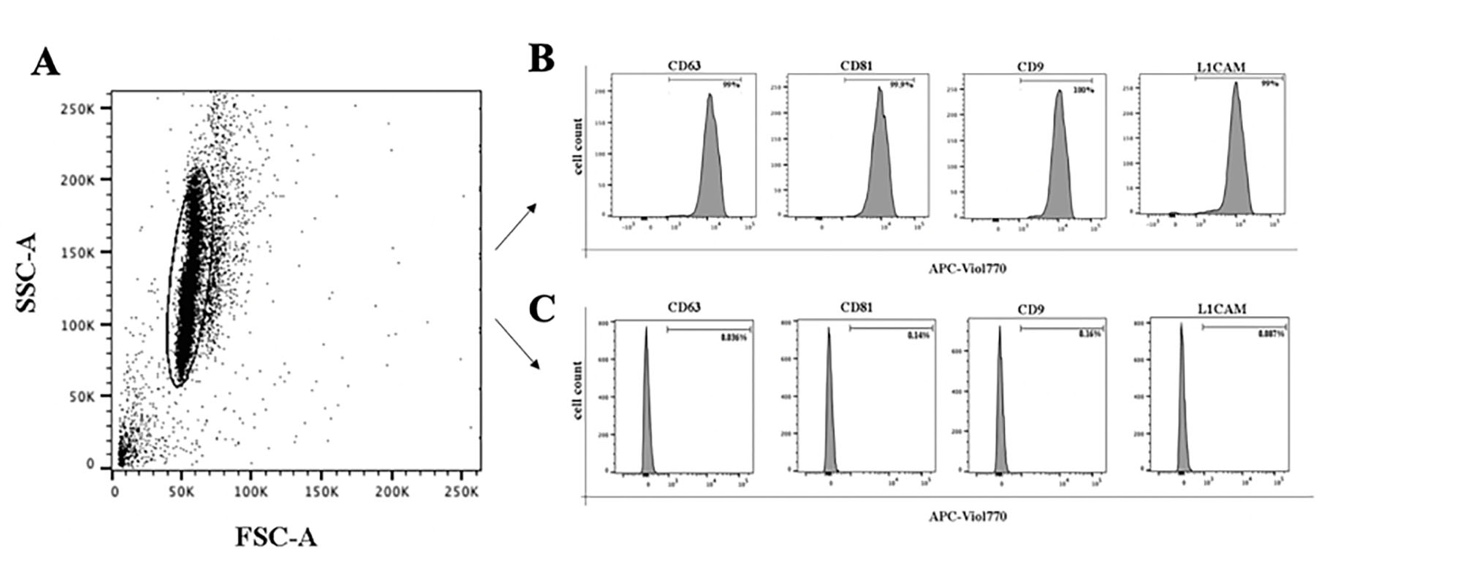
**

**Supplementary Figure S1:** Flow cytometry on NDEVs decorated beads (A) showed the expression of tetraspanines (CD63, CD9, CD81) and L1CAM in NDEVs (B), while no EVs were isolated using biotin-conjugated isotype antibody (C).
