# Supplementary figures and images for: Neurofilament Light Chain from Neuronally Derived Extracellular Vesicles in Differentiating Parkinson’s Disease from Essential Tremor with Resting Tremor
Source: Mol Neurobiol. 2025 Nov 11;63(1):24. doi: 10.1007/s12035-025-05285-7 (PMC12605399; doi:10.1007/s12035-025-05285-7)

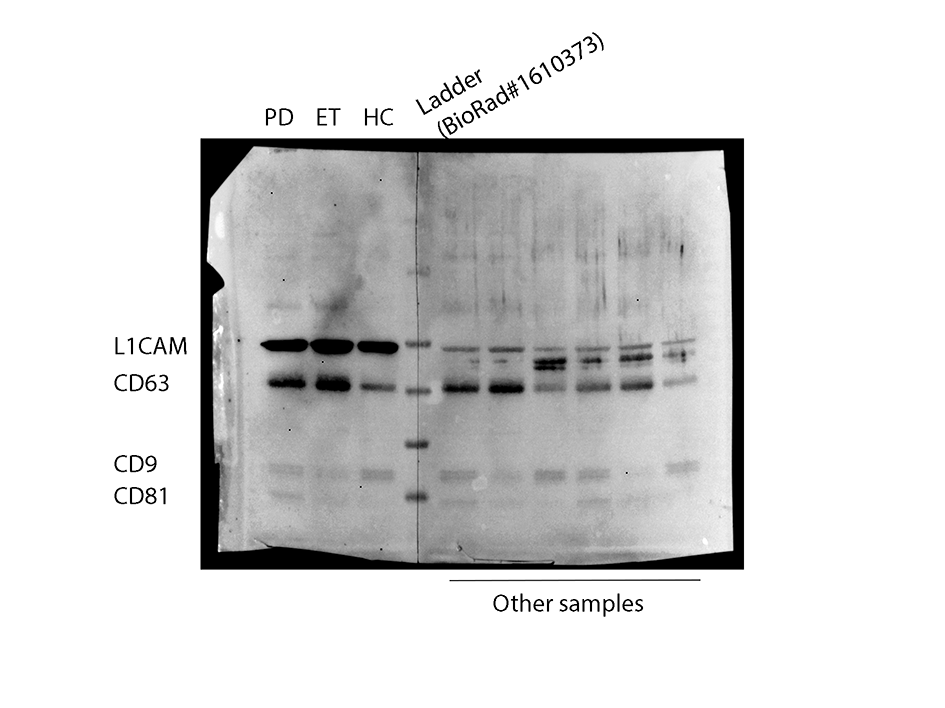

Supplement: Supplementary file 2 — Supplementary 2 (PNG 196 KB) [file 12035_2025_5285_Fig4_ESM.png]

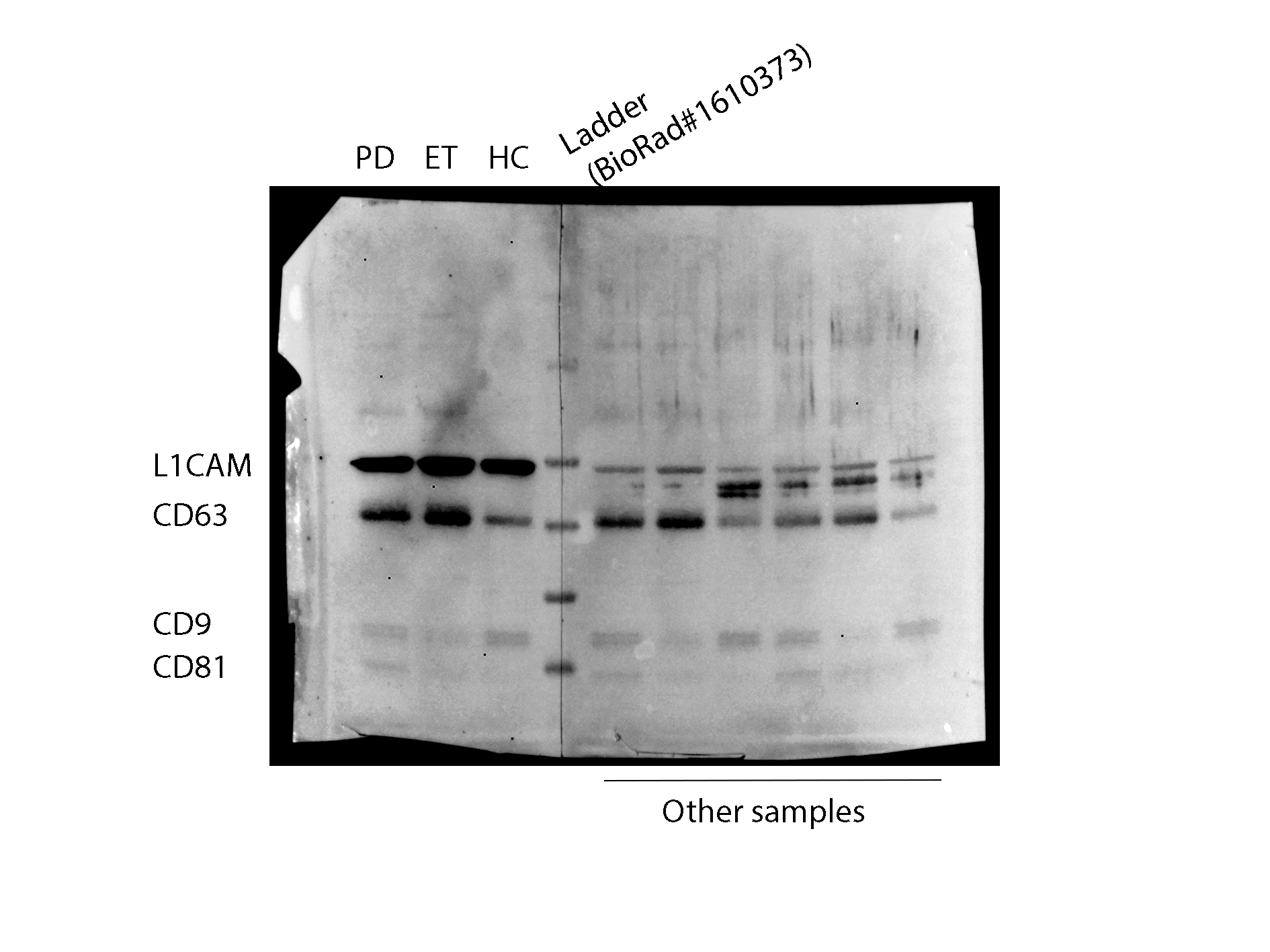

Supplement: Supplementary file 3 — High Resolution Image (TIF 10.4 MB) [file 12035_2025_5285_MOESM2_ESM.tif]
